# Reliable detection of RNA in hippocampus sections of mice by FISH up to a post-mortem delay of 24 h

**DOI:** 10.1007/s00418-024-02277-x

**Published:** 2024-04-06

**Authors:** Sophie Seiffer, Jana Brendler, Angela Schulz, Albert Ricken

**Affiliations:** 1 Institute of Anatomy, Medical Faculty, Leipzig University, Leipzig, Germany; 2 Medical Faculty, Rudolf Schönheimer Institute of Biochemistry, Leipzig University, Leipzig, Germany

**Keywords:** FISH, RNA, Post-mortem delay, Mouse, Brain, Hippocampus, Microglia, Gpr34, P2ry12, Housekeeping genes

## Abstract

Proteins can be successfully localized in post-mortem (PM) brain tissue sections if the time until PM tissue sampling is not too long. In this study, we show that this also applies to the localization of RNA and in particular to the RNA of microglia-specific receptor proteins using the probes and the RNAscope^™^ Multiplex Fluorescent Detection Kit v2 from Advanced Cell Diagnostics. Brains were removed from killed mice after different PM delays and processed into paraffin sections. In sections of brains from animals whose cadavers had been kept at room temperature (21 °C) before tissue removal, ubiquitously expressed RNAs of genes with low to high expression levels (Polr2a, PPIB, and UBC) were reliably detected in the brain sections even if tissue removal was delayed by up to 48 h. In addition, microglia-specific G protein-coupled receptor RNA (Gpr34, P2ry12) could be reliably assigned to microglia by simultaneous labeling of the microglia with microglia-specific antibodies (Iba1 or P2ry12). Only after a delay of 48 h until tissue removal were the receptor RNA signals significantly lower. The reduction in receptor RNA signals could be delayed if the animal cadavers were stored at 4 °C until the brains were removed. Tissue sections of PM brain samples allow the spatial and cellular localization of specific RNA, at least if the sampling takes place within the first 24 h of PM.

**Supplementary Information:**

The online version contains supplementary material available at 10.1007/s00418-024-02277-x.

## Introduction

The healthy human brain can be examined externally by using devices or analyzing the draining blood and cerebrospinal fluid. Pathological or traumatic changes can also be examined to a limited extent on resection specimens taken during surgery. The histological examination of healthy and pathological human brain tissue is at best accomplished by examining the brain tissue of deceased donors (ElHajj et al. 2016; Winkelmann 2016).

The delay between death and autopsy in humans varies significantly: hours or days can pass under very different climatic conditions until the brain tissue can be properly removed, preserved and processed. This is known as post-mortem (PM) delay (Heng et al. 2021; Scholefield et al. 2020). Consequently, when examining brain tissue samples, PM tissue changes at the molecular, immunological, structural and functional level must inevitably be accepted and considered when collecting pathological-anatomical, physiological and biochemical data from the material. In addition, changes in brain cells that have occurred prior to death because of a chronic illness or in the dying process (e.g., coma, hypoxia, fever, dehydration and medication) must be considered (Durrenberger et al. 2010; Groot et al. 1995; Heng et al. 2021; Stan et al. 2006). Within minutes of irreversible cessation of cardiopulmonary function, the brain is irreparably damaged because of the lack of oxygen supply (van Lommel et al. 2001). PM decomposition comprises the steps of autolysis, putrefaction and decay. The outcome of the first two steps depends on the climatic conditions under which the cadaver is stored until the brain tissue is removed and preserved as well as on the cell population subsequently considered, e.g., glial and/or neuronal cells (Dachet et al. 2021; MacKenzie 2014).

For these reasons, laboratory animals, particularly rodents, are used for neuroscientific studies. The brains can be selectively removed and processed in a standardized way. Findings however are limited regarding their transferability to humans (Dachet et al. 2021; ElHajj et al. 2016; Yvanka de Soysa et al. 2022). Identical experimental set-ups with healthy, age- and sex-matched inbred mice housed under standardized conditions allow for experimental treatments to be carried out in a controlled manner. Animals with defined genetic modifications can be studied under controlled conditions as well (Groot et al. 1995).

In most cases, the brains of laboratory animals are removed immediately after the animals have been killed and are further processed for the morphological and molecular biological examinations. The results obtained with these brains are then often compared with the histological and/or molecular biological characteristics of brains from human donors, without considering the pre- and PM changes to which these human brains were exposed (see above). It is therefore of interest to investigate the influence of the PM delay on the brains of laboratory animals and to examine the extent to which the PM changes still allow a visualization and characterization at the tissue and cell level.

It is known that brain sections from rodents and humans, which are sometimes removed from cooled bodies with a PM delay of up to 2 months, can be suitable for immunohistochemical and immunofluorescence (IF) studies in addition to their molecular biological usability (Dachet et al. 2021; Gelpi et al. 2007; Groot et al. 1995; Hilbig et al. 2004).

We are investigating the usability of brain sections from mice with different PM delays for RNA FISH detection by laser scanning microscopy. Additionally, the feasibility of co-detecting specific proteins for cell visualization was tested in the brain tissue. We selected the hippocampus (HC) and its microglia for the study because of their significant involvement in the pathophysiology of Alzheimer's disease in humans (Streit 2004). In the vicinity of the deposits associated with Alzheimer's disease, i.e., neurofibrillary tangles and β-amyloid, microglia switch from a branching, resting phenotype to an amoeboid, phagocytic phenotype and produce a cascade of pro-inflammatory cytokines (Lier et al. 2021). P2ry12 and Gpr34 are two G protein-coupled receptors (GPCRs) in the plasma membrane of microglia that are involved in the activation process and are used as microglia markers in many studies (Maeda et al. 2021; Olah et al. 2020; Schöneberg et al. 2018; Walker et al. 2020). Our study serves as a methodological basis for the use of the FISH technique in addition to IF labeling in the examination of tissue sections from the brains of Alzheimer’s patients with different PM delays in tissue removal and preservation.

## Materials and methods

For the study, 1.8-mm coronal brain sections at the bregma were used from a total of 32 3-month-old healthy male C57BL/6 WT [five mice for each of the four time points in the 21 °C room temperature (RT) experiments and three mice for each of the four time points in the 4 °C experiments; for details, see below] as well as from four 3-month-old male Gpr34-deficient C57BL/6 mice processed without PM delay (Liebscher et al., 2011). Approval was given by the respective regional animal welfare agency of the state of Saxony, Germany (T21/18). The mice used in the study were bred on site under standard conditions (12 h day/night cycle, free access to water and food, two to five animals per cage) and were killed for brain extraction by euthanasia with CO_2_. Depending on the desired PM delay, the brains were removed from the cadavers immediately or after 12, 24 or 48 h of storage in an air-conditioned laboratory at a constant room temperature (RT) of 21 °C (five brains per time point) or at 4 °C in a refrigerator (three brains per time point). Then, the HC was removed, fixed in 4% formaldehyde solution in phosphate-buffered saline with a pH of 7 (Roti^®^Histofix 4%, Carl Roth, Karlsruhe, Germany) for 24 h at 4 °C over night and embedded in paraffin with a melting point at 56 °C (Rot^i®^Plast, Carl Roth). Frontal sections (10-µm-thick) were prepared from the paraffin blocks in the rostral-caudal direction and mounted on microscope slides. The sections were deparaffinized, rehydrated and HE stained. Subsequent sections with comparable histological regions were selected for further analysis. From each series of HC sections, at least three follow-up sections were selected and experimentally analyzed as described below. For each experiment, at least one section of the appropriate HC specimen was used.

### Single molecule RNA detection of housekeeping gene RNA targets by fluorescent in situ hybridization (FISH)

We used the RNAscope^™^ Multiplex Fluorescent Detection Kit v2 from Advanced Cell Diagnostics (ACD), a brand of Bio-Techne GmbH (catalog no. 323110, Bio-Techne GmbH, Wiesbaden-Nordenstadt, Germany) according to the manufacturer's instructions to separately detect several housekeeping gene RNA targets in one section as single molecules. The probes used were contained in the mouse-specific ACD RNAscope three-plex positive control probe mixture Mm (no. 320881, Bio-Techne GmbH) and were directed against the RNA of mouse Pol2a (RNA polymerase II subunit A; channel C1), PPIB (peptidyl-prolyl isomerases B; channel C2) and UBC (ubiquitin C, channel C3). The linker system included in the kit and the recommended fluorescent dyes from Akoya Biosciences (Marlborough, MA, USA) were used to visualize the sample bindings in the indicated dilution range, i.e., Opal 520 reagent (part no. FP1487001KT) for channel C1, Opal 570 reagent (part no. FP1488001KT) for channel C2 and Opal 690 reagent (part no. FP1497001KT) for channel C3; all were diluted 1:750. As a negative control, we used the ACD RNAscope^®^ three-plex negative control probe mixture (catalog no. 320871, Bio-Techne GmbH), which contains a mixture of C1, C2 and C3 samples against the bacterial RNA of the DapB (4-hydroxy-tetrahydrodipicolinate reductase) gene, to estimate the background noise and interpret the results obtained with the assay. Zeiss LSM 700 confocal laser scanning microscopy (Carl Zeiss, Oberkochen Germany) was used to evaluate and document the results.

### Single-molecule RNA detection of microglia-specific RNA targets by FISH in combination with microglia labeling by IF

First, we used the RNAscope^™^ Multiplex Fluorescent Detection Kit v2 as described above to detect microglia-typical intracellular RNA targets. We used either a P2ry12- or Gpr34-specific RNA probe from ACD (catalog no. 317601-C3 or 318201, Bio-Techne GmbH) and Opal 570 reagent as fluorochrome. Following the RNAscope^™^ Multiplex Fluorescent Detection Kit v2 assay with the microglia-specific RNA probes, IF visualization of microglia was performed with two well-characterized microglia markers. In one approach, we used a 1:100 dilution of polyclonal P2ry12 antibody from Sigma (catalog no. HPA014518, Taufkirchen, Germany) against the GPCR P2ry12, which is expressed on the cell surface of microglia and corresponded to the P2ry12 RNA probe (Shaik et al. 2023). For the other approach, we used a 1:100 dilution of the recombinant monoclonal guinea pig Iba1 antibody from Synaptic Systems (catalog no. 234 308, Göttingen, Germany) against the allograft inflammatory factor 1 in the cytoplasm of the microglia (Glotfelty et al. 2023; Riemenschneider et al. 2023). To detect the primary antibody binding, we used an Alexa 488-coupled secondary antibody from Invitrogen (A11034 and A11073, Darmstadt, Germany) matching the species of the primary antibodies. Again, the cell nuclei were routinely counterstained with DAPI, and Zeiss LSM 700 confocal laser scanning microscopy was used to evaluate and document the results of the FISH/IF co-analyses. The FISH/IF co-detection was accompanied by control experiments in which the microglia-specific antibody was omitted. No staining resembling the visualization of microglia with the specific antibody was seen in the sections of the control experiments (not shown).

## Results and discussion

We present a technical study on the feasibility of detecting RNA from differentially expressed genes by FISH in mouse HC tissue collected and processed immediately after death or after storing the cadavers for up to 48 h at 21 °C RT or at 4 °C. For this, we used the commercially available ACD RNAscope^™^ Multiplex Fluorescent Detection Kit v2.

### Determination of the PM influence on the detectability of ubiquitously expressed gene products in mouse HC sections

**Fig. 1:**
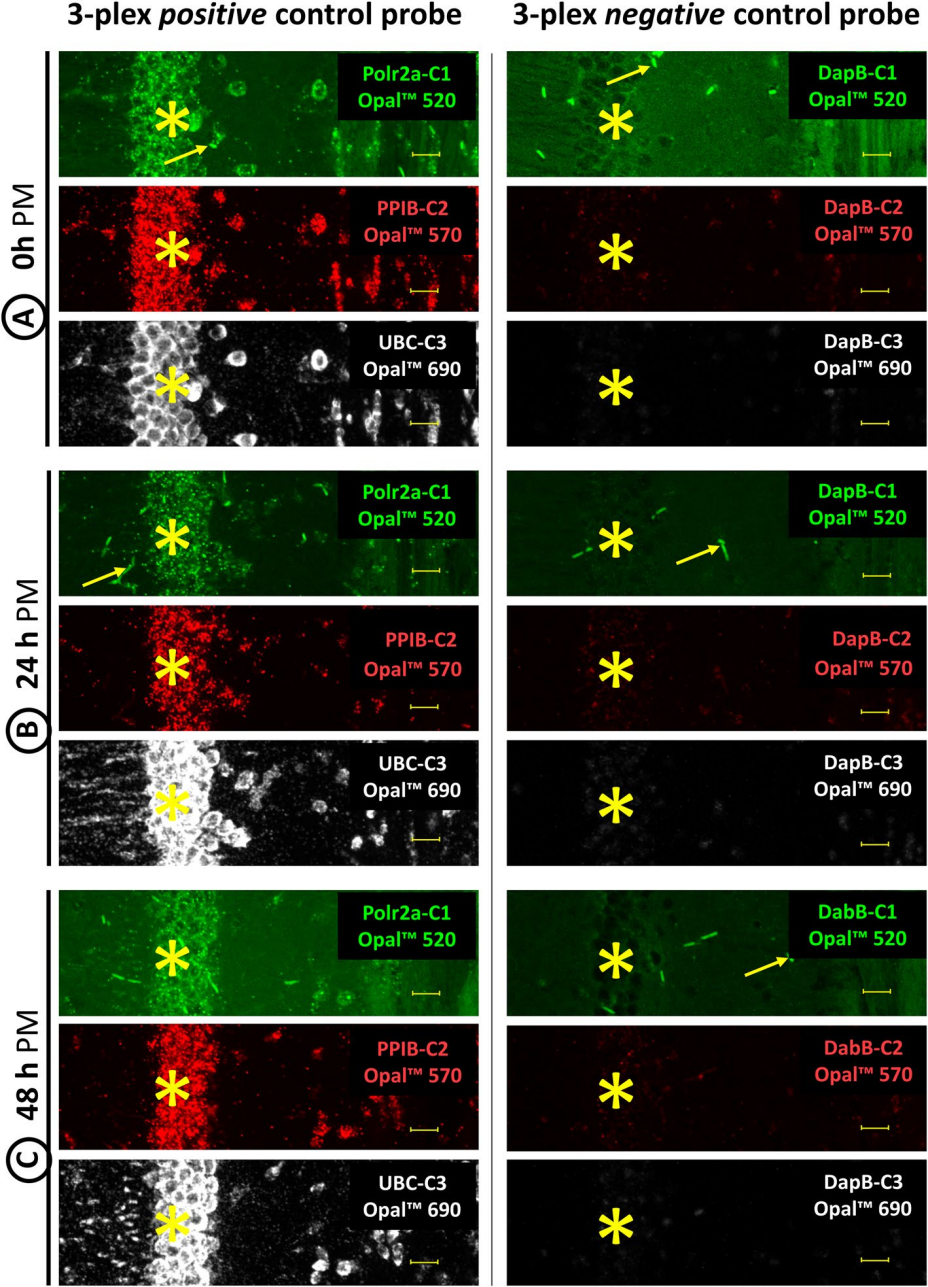
Detection of the expression of housekeeping genes with different expression levels by FISH in HC sections of samples with different PM delays (**A**: 0 h, **B**: 24 h and **C**: 48 h) until sampling (left column). Compared to experiments with the negative control probe against bacterial DapB (right column), the positive control probe, consisting of an oligo mix against Polr2A (low expression level, green), PPIB (medium expression level, red) and UBC (high expression level, white), leads to reliable positive signals in the HC sections with 0 h, 24 h and even 48 h PM delay. The specific signals can be easily distinguished from non-specific background signals, which mainly originate from blood vessels (arrows) in the sections. The asterisks indicate the position of the pyramidal cell layer of the CA field with low background staining. Bars equal 20 µm

The extent to which RNA remains detectable in HC sections with increasing PM delays was initially investigated in experiments using a positive control probe mixture directed against RNAs of ubiquitously expressed genes (Fig. 1 left column). Thereby, the positive control probe consisted of an oligo mix against the RNA of genes with low (Polr2a, C1-Opal 520, green signal), medium (PPIB, C2 channel, C2-Opal570, red signal) and high (UBC, C3-Opal 690, white signal) expression rate according to the manufacturer's specifications. The results obtained with the positive control probe were compared with results using a negative control probe with an oligo mix against bacterial DapB RNA in all channels (Fig. 1 right column). FISH labeling with the positive control probe but not with the negative control probe resulted in cellular bound signals condensing in the pyramidal cell layer of the cornu ammonis (CA) field (Fig. 1, left column, asterisks). The staining patterns elicited by the oligo mix for Polr2a (C1), PPIB (C2) and UBC (C3) of the positive control probe mixture in the different amplification and visualization approaches (C1-Opal 520, green; C2-Opal570, red; C3-Opal 690, white) were almost identical.

When comparing signals in sections of HC tissue preserved without PM delay with signals obtained in sections of PM-delayed preserved HC tissue, RNA detection with the positive control probe for housekeeping genes remained reliable for 24 h (Fig. 1B) and 48 h (Fig. 1C) PM delay brain tissue sections, with impressive specificity (lack of significant signal response with the negative control probe) and sensitivity (detection of RNA from the Polr21 gene with low expression). The strength of the Polr2a and PPIB signal in sections of samples with different PM delays appeared to be comparable, while the UBC signal appeared to increase in strength with longer PM delay times.

In the experiments with the control probes, mainly unspecific fluorescence signals were observed, which originated from small vessels (Fig. 1, arrows). Since these non-specific fluorescence signals were already visible in the sections of brain tissue preserved without PM delay (Fig. 1 A) and occurred regardless of whether FISH probing was performed with the positive (Fig. 1 left column) or negative (Fig. 1 right column) control probes, tissue- or detection system-related background staining is more likely than non-specific binding of the probes in addition to their specific binding.

### Simultaneous visualization of microglia with antibodies and detection of microglia RNA in HC sections of different PM delay brain samples

**Fig. 2:**
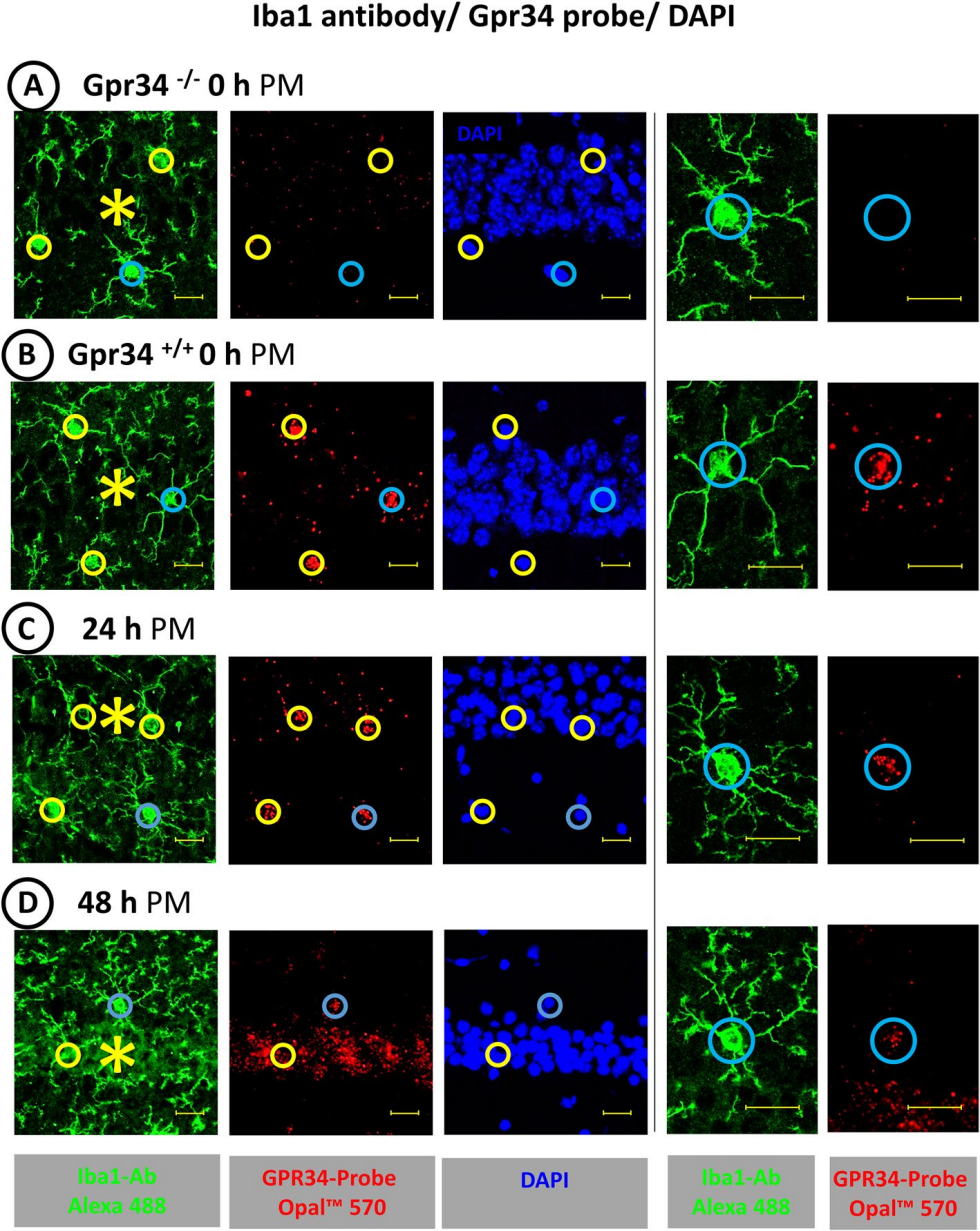
FISH/IF labeling of microglia in HC sections from brains with a PM delay of **A** and **B**: 0 h, **C**: 24 h and **D**: 48 h. In contrast to HC sections from Gpr34-deficient mice with 0 h PM delay (**A**), co-labeling of wild-type HC microglia with a Gpr34 probe by FISH (red) and an Iba1 antibody by IF (green) with **B**: 0 h, **C**: 24 h and **D**: 48 h PM delay results in congruent localization of microglia-specific signals. In the HC section with 48 h PM delay, the assignment of the remaining Gpr34 FISH signal to the Iba1-labeled microglia is just possible. The colored circles enclose the green Iba1 IF-labeled microglia cell bodies and their red Gpr34 RNA signals; the blue circle indicates the enlarged cell. The asterisks indicate the position of the pyramidal cell layer of the CA field. Bars equal 20 µm

**Fig. 3:**
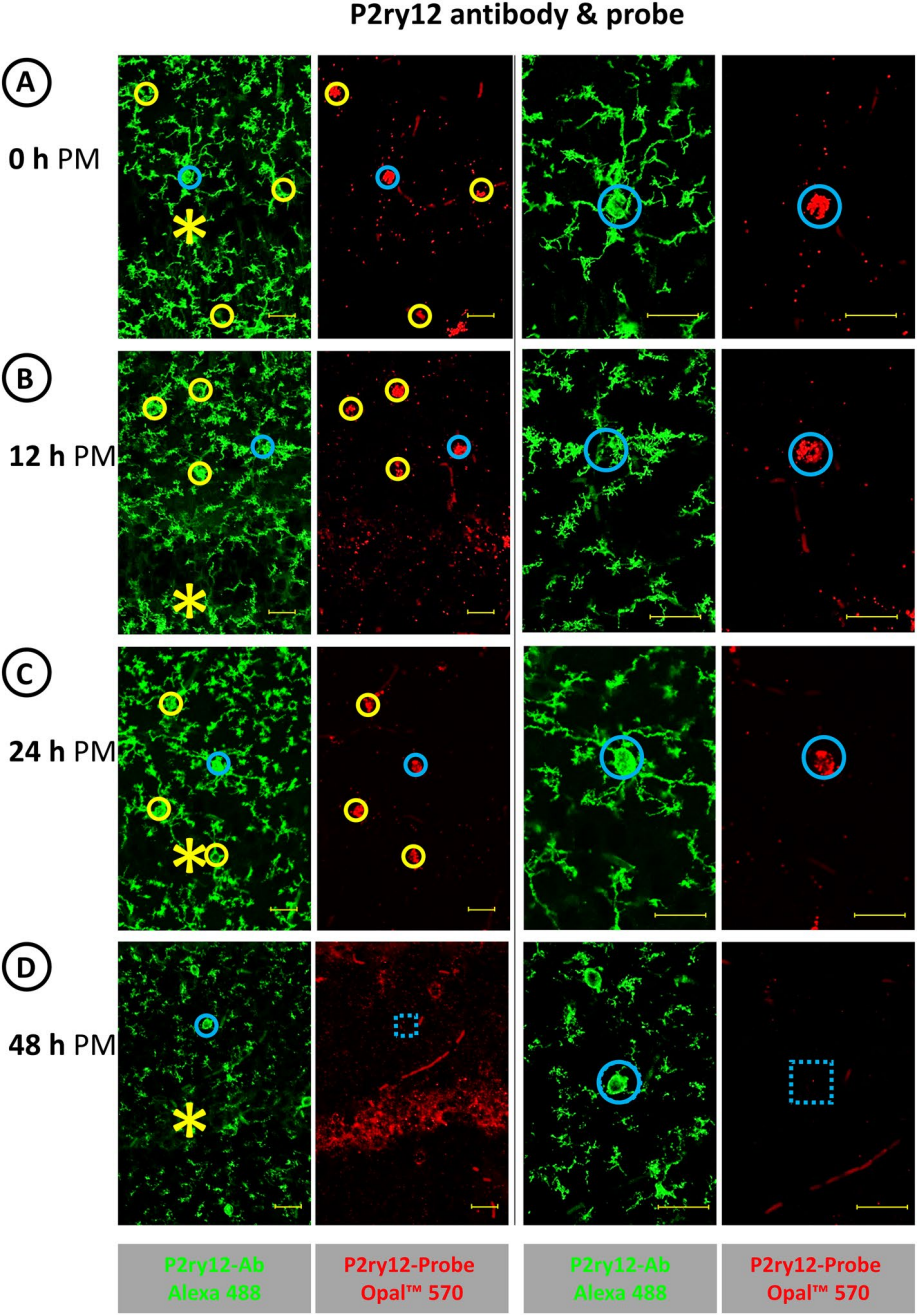
Matching labeling of microglia with a P2ry12 RNA probe (red) and a P2ry12 antibody (green) in HC sections from samples with a PM delay of up to 24 h (**A**–**C**). **D** In HC sections with a delay of 48 h until sampling, an assignment of a P2ry12 FISH signal to the antibody marked microglia is no longer possible. Note the change in shape of the microglia from a branched type in the 0 h PM delay brain (**A**) via a fuzzy looking type (**B** and **C**) to a type with apparently discontinuous processes in the 48 h PM delay (**D**) brain. The colored circles enclose the green P2ry12 IF-labeled microglia cell bodies and their red P2ry12 RNA signals; the blue circle indicates the respective enlarged cell. The interrupted square indicates the region in which the positive signal would have been expected. The asterisks indicate the pyramidal cell layer of the CA field. Bars equal 20 µm

The ACD RNAscope™ Multiplex Fluorescent Detection Kit v2 is designed so that the RNA of interest triggers a signal if oligos from the oligo pool bind to two immediately neighboring sequences of the RNA of interest. Thus, the detection method achieves a high specificity, which we verified when we analyzed HC sections from Gpr34-deficient and wild-type mice using a Gpr34 probe in combination with IF labeling of microglia with a microglia-specific Iba1 antibody. In contrast to the lack of RNA/protein co-detection in HC sections from the Gpr34-deficient mice (Fig. 2A), FISH with the Gpr34 probe (red) and IF labeling with the Iba1 antibody (green) resulted in matching staining of microglial cell bodies in HC sections from wild-type mice (Fig. 2B). Up to a PM delay of 24 h, the Gpr34 RNA signal of the microglial cell bodies stood out from any nonspecific background staining because of its high intensity (Fig. 2C). Thus, up to a PM delay of 24 h at 21 °C RT, recognition of specific GPCR RNA labeling was not hampered by background staining, e.g., from small blood vessels (see above). However, at 48 h PM delay, a nonspecific background staining, arising from the pyramidal cell layer of the CA field (Fig. 2D) and the granular layer of the dentate gyrus (not shown), made it difficult to assign the existing Gpr34 signals to Iba1 IF-labeled microglia. The parallel performed detection of RNA for the microglia-typical GPCR P2ry12 (red) in combination with the P2ry12 IF labeling of microglia (green) in Fig. 3 demonstrated a similar detection level of GPCR RNA in PM delay HC sections as the combined detection of Gpr34 RNA and Iba1 protein. Here, too, the RNA signals obtained by FISH could be clearly assigned to the IF-labeled microglial cell bodies up to a PM delay of 24 h. In contrast to the approaches with the Gpr34 probe, no P2ry12 RNA signals were detectable in the still P2ry12-immunolabeled microglia in the 48 h PM delay HC sections. In summary, using the ACD RNAscope™ Multiplex Fluorescent Detection Kit v2, RNAs for the microglial proteins Gpr34 and P2ry12 are readily detectable by FISH in HC sections up to a PM delay of 24 h at 21 °C RT until tissue removal and preservation; after a PM delay of 48 h at 21 °C RT, however, the RNA signals are significantly reduced or absent, although the microglia can still be localized by Iba1 or P2ry12 antibody IF labeling.

### Influence of the PM storage temperature on RNA detection by FISH

**Fig. 4:**
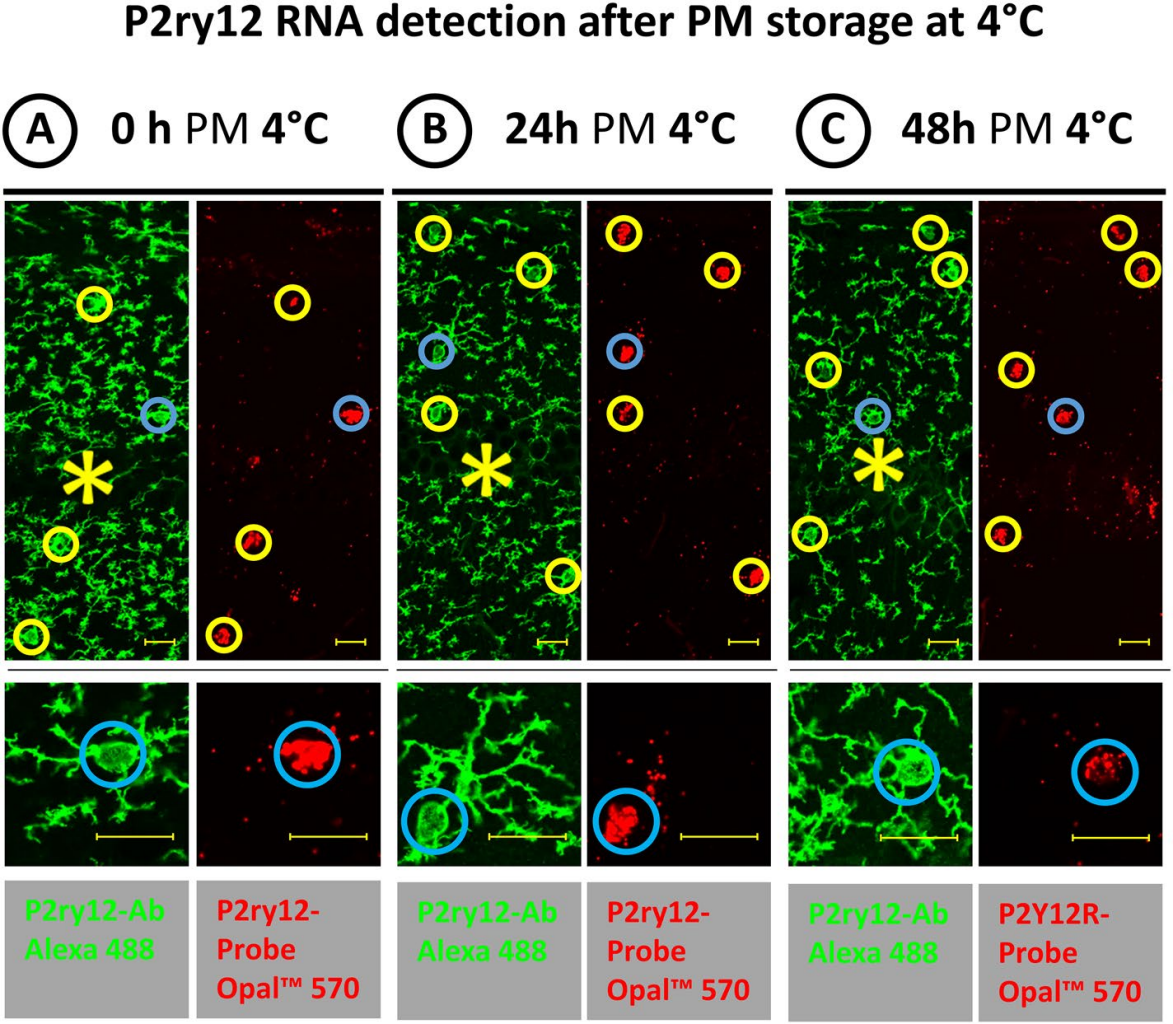
Effects of temperature until PM removal and preservation of brain samples on the detection of RNA in mouse HC sections with different PM delays until sampling (**A**: O h, **B**: 24 h, **C** 48 h). Chilling the animal cadavers to 4 °C prolongs the detectability of RNA in brain tissue. In particular, microglia-specific P2ry12 RNA can still be detected with sufficient reliability in 48 h PM delay brain samples (**C**) when the animal cadavers are stored at 4 °C compared to room temperature until sampling (Fig. 3D). The colored circles enclose the green P2ry12 IF-labeled microglia cell bodies and their red P2ry12 RNA signals; the blue circle indicates the respective enlarged cell. The asterisks indicate the pyramidal cell layer of the CA field. Bars equal 20 µm

Both the detection of ubiquitously expressed genes in the HC sections and the detection of microglia-specific gene products benefited from the storage of the cadavers at 4 °C instead of a constant 21 °C until sampling. When animal cadavers were stored at 4 °C, the three different housekeeping RNA targets and the microglia-specific RNA targets Gpr34 and P2ry12 were readily detectable in HC sections of samples with a 48 h PM delay, as shown for P2ry12 in Fig. 4. This is in agreement with human studies suggesting that the rate and manner in which bodies and heads are cooled after death affect the integrity of RNA in the brains (Ervin et al. 2007).

### Phenotypic change of PM microglia until tissue removal and preservation for microscopic sectioning

Interestingly, IF labeling of microglia with the two cell-type-specific antibodies Iba1 and P2ry12 revealed a change in the phenotype of microglia depending on the PM delay until the samples for HC sections were obtained. With constant storage of the animal cadavers at 21 °C RT, the microglia changed with increasing PM time from a branched type with few secondary processes (at immediate sampling, Fig. 3A) to a fuzzy-looking type with many secondary processes (at sampling after 12 and 24 h PM, Fig. 3B and C) and finally to a phenotype with discontinuous presentation of its processes (at sampling after 48 h PM, Fig. 3D). A phenotypic change in microglia with increasing PM delay until tissue removal and preservation was also observed in HC sections from cadavers stored at 4 °C, albeit to a lesser extent over the time span studied. In the HC sections from cadavers stored for 24 h and 48 h at 4 °C, microglia with numerous secondary processes were observed; however, in contrast to the HC sections from cadavers stored for 48 h at 21 °C, no microglia with apparently discontinuous processes were observed in the HC sections from cadavers stored for 48 h at 4 °C (see the enlargements in Fig. 4B and C).

## Conclusion

Our results complement and extend studies on freshly isolated microglia showing that vital microglia with high-quality RNA and only minor changes in RNA expression can still be isolated from brains up to 12 h or more PM (Heng et al. 2021). They are also consistent with studies reporting a small effect of PM delay on overall RNA quantity in PM brain tissue (Barton et al. 1993; Durrenberger et al. 2010; Kobayashi et al. 1990) and that neurons and glia cells in cultured PM brain tissue slices can remain viable for weeks and are capable of expressing genes de novo, e.g., virus-transfected ones (Verwer et al. 2002a, 2002b). However, that the expression of genes for glial activity may persist or even increase after a PM delay of > 4 h, while the PM expression of genes for neuronal activity may decrease rapidly, must be considered (Dachet et al. 2021). Although the focus of our work is on microglia-specific RNA probes, the strong signal obtained with the positive control probe against the RNA of differentially expressed housekeeping genes in the pyramidal cell layer of the CA field suggests that results comparable to our microglia-specific RNA probes can also be obtained with at least some neuron-specific RNA probes. Furthermore, although we performed our studies only on HC sections, it can be assumed that only marginal differences in the PM delay results would arise if brain cells, especially microglia, were examined in other brain regions, as shown by protein and RNA analyses of PM human brains by others (Hilbig et al. 2004; Stan et al. 2006). Indeed, we obtained comparable FISH-IF results in other brain regions (cortex, habenula, hypothalamus) when we analyzed sections from 24 h PM brain samples with the P2ry12 probe (supplemental Fig. 1).

The changes in the microglia phenotype could indicate PM changes in the microglia that cannot be explained by the onset of tissue decay alone. We observed a change in the shape of the microglia from a branched type with few secondary processes to a fuzzy-looking type with many secondary processes and finally to a microglia type with discontinuous presentation of its processes. In addition to the PM onset of organ and tissue decay and the resulting chemical and structural changes, the observed changes in microglia phenotype could also be related to PM activation of microglia caused by the PM changing environment, e.g., reduced oxygen levels (Dachet et al. 2021; Hilbig et al. 2004). Changes in the shape and Gpr34/P2ry12 gene expression have been described in the activation of microglia, which occurs in numerous neurodegenerative diseases such as Alzheimer’s disease (Haque et al. 2018; Lier et al. 2021; Schöneberg et al. 2018). However, in microglia isolated from PM brains, the expression of only a few genes is altered by the PM delay (Heng et al. 2021). The altered microglia genes are mainly genes responsible for mitochondrial, ribosomal and protein-binding functions (Heng et al. 2021). In the positive control probe experiments, we found that the signal for the gene with the highest expression level of the three oligos, UBC, appeared even to be increased in the HC sections with the longer PM delay. This observation is consistent with reports of prolonged stable and possibly even increased PM activity of the lysosomal and ubiquitin-proteasome systems in neuronal and other tissues (ElHajj et al. 2016; Ferrer et al. 2007; Lamare et al. 2002; Orre et al. 2013).

The FISH results for 21 °C RT indicate that, after a PM delay of 48 h at RT, structural and chemical events occur that make the brain tissue no longer fully suitable for the in situ detection of RNA by the FISH technology used in the study. Prior to this, such detection appears to be feasible, although again, as at the protein level, that some RNA species may be more susceptible to degradation or expression changes than others must be considered (ElHajj et al. 2016; Hilbig et al. 2004; Stan et al. 2006). Our observation that the microglia-specific RNA in the HC sections appeared to be partially degraded after 48 h PM, while the associated proteins were still detectable, is consistent with molecular biological tissue studies by others (Stan et al. 2006). However, it is conceivable that with improved technologies, for instance, enhanced dyes, lower background and/or improved storage conditions, FISH could reliably detect RNA signals at later PM delay times than in this study.

In summary, our study addresses a methodological question by showing that PM brain tissue sections can be used not only for IF labeling but also for FISH. We demonstrate this using HC sections from mouse brains with a PM delay of up to 24 h at 21 °C RT until sampling. Up to this PM delay time, the specific RNA signals exceed non-specific background signals so that they can be reliably distinguished. The validity of FISH on brains with a longer PM delay can be increased by storing the cadavers at lower temperatures, e.g., 4 °C, until sampling. It can be hypothesized that with improved technologies, for instance, improved dyes, lower background and/or improved storage conditions, FISH could detect RNA signals at later PM delay time points than in our study. Our work also supports findings suggesting that microglia undergo a transient activation process after death until their final decomposition.

### Supplementary Information

Below is the link to the electronic supplementary material.

Supplementary file1 (TIF 30244 KB)

## Data Availability

Samples and data used to generate the results in the paper are available on request.
