# Supplementary figures and images for: Reliable detection of RNA in hippocampus sections of mice by FISH up to a post-mortem delay of 24 h
Source: Histochem Cell Biol. 2024 Apr 6;161(6):539–47. doi: 10.1007/s00418-024-02277-x (PMC11162364; doi:10.1007/s00418-024-02277-x)

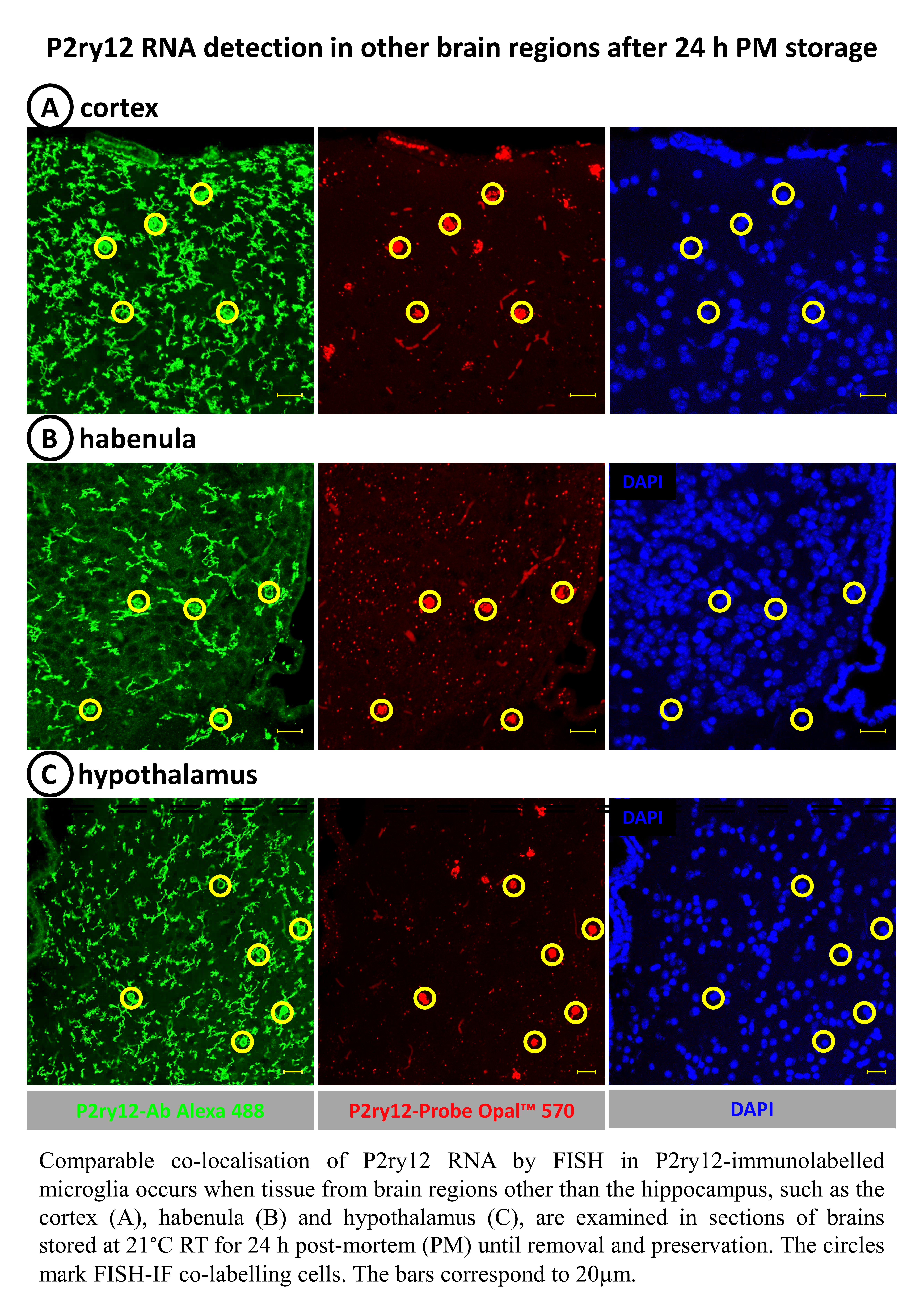

Supplement: Supplementary file 1 — Supplementary file1 (TIF 30244 KB) [file 418_2024_2277_MOESM1_ESM.tif]
